# Supplementary material for: Prevalence of food insecurity amid COVID-19 lockdowns and sociodemographic indicators of household vulnerability in Harar and Kersa, Ethiopia
Source: BMC Nutr. 2024 Jan 9;10:7. doi: 10.1186/s40795-023-00815-9 (PMC10777627; doi:10.1186/s40795-023-00815-9)
Supplement: Supplementary file 1 — Additional file 1: Supplementary Methods. Wealth Index Generation. Supplementary Methods. Survey Instrument. [file 40795_2023_815_MOESM1_ESM.zip › COVID_CHAMPS_Instrument.pdf]

RESPONDENT NUMBER: __ __ __ __ __ __

Harmonized COVID-19 Impact Questions for the CHAMPS HDSS Network

Household DSS ID: ____________ Individual DSS ID: ____________

Interviewer's name: ________________ Interviewer's code: __ __ __

Interview date: __ __/__ __/__ __ __ __

Interview start time: __________________

INTERVIEWER READ THE FOLLOWING INFORMATION:

(ADD HERE STATEMENT OF THE PURPOSE OF THE STUDY, USE OF DATA, CONFIDENTIALITY, SPONSORS, ETC. AS APPROPRIATE)

INSTRUCTION FOR INTERVIEWER

1. INSTRUCTIONS HAVE BEEN PROVIDED IN CAPITAL LETTERS. DO NOT READ THESE INSTRUCTIONS TO RESPONDENTS.
2. READ ALL QUESTIONS AS WRITTEN. DO NOT READ RESPONSES UNLESS INSTRUCTIONS EXPLICITLY STATE TO DO SO.
3. IF QUESTION INSTRUCTIONS STATE TO READ RESPONSE, DO NOT READ "DON'T KNOW" OR "REFUSED". CIRCLE THESE ONLY IF THEY ARE THE ANSWER GIVEN BY THE RESPONDENTS.
4. INDICATE THE RESPONSE THAT CORRESPONDS TO THE RESPONDENT'S ANSWER.
5. FOR OPEN QUESTION, ENTER THE RESPONDENT'S ANSWER ON THE SPACE PROVIDED.

RESPONDENT NUMBER: __ __ __ __ __ __

Knowledge Regarding the Spread of COVID-19

INTERVIEWER TO PARTICIPANT: I would like to ask **the head of the household or their spouse** some questions about coronavirus or COVID-19

V	Question	Response	Skip
V1	**Have you heard about the coronavirus/ COVID-19?** (CIRCLE ONE RESPONSE)	Yes....1<br>No....2<br>Don't know....98<br>Refused to respond....99	 ->P<br>->P<br>->P
V2	**To your knowledge, which of the following measures can you adopt to reduce the risk of contracting coronavirus/COVID-19?** (PLEASE DO NOT READ, CHECK MULTIPLE RESPONSES THAT APPLY, OR WRITE RESPONSE FOR 88)		

	No (00)	Yes (01)	Don't know (98)	Refused to Respond (99)
1. Handwashing	☐	☐	☐	☐
2. Sanitizer use	☐	☐	☐	☐
3. Avoid handshake/physical greeting	☐	☐	☐	☐
4. Mask use	☐	☐	☐	☐
5. Gloves use	☐	☐	☐	☐
6. Avoid travel	☐	☐	☐	☐
7. Staying at home	☐	☐	☐	☐
8. Avoid going out unless necessary	☐	☐	☐	☐
9. Avoid crowded places	☐	☐	☐	☐
10. Keep ~2 meters space between yourself and others	☐	☐	☐	☐
11. Other, specify (88)	______________			

RESPONDENT NUMBER: __ __ __ __ __ __

V3	**What steps has your community/government taken to curb the spread of the coronavirus in your area?**<br><br>(PLEASE READ ALOUD, SELECT ALL THAT APPLY)	None......0<br>Advised citizens to stay at home......1<br>Advised to avoid gatherings......2<br>Restricted travel within country/area......3<br>Restricted international travel......4<br>Closure of schools and universities......5<br>Curfew/lockdown......6<br>Closure of non-essential businesses......7<br>Sensitization/public awareness......8<br>Established isolation centers......9<br>Disinfection of public places......10<br>Other......88<br>Don't know......98<br>Refused to respond......99	
	**Other, specify**	______________	
V4	**Were any of your household members tested for COVID-19?**<br><br>(CIRCLE ONE RESPONSE, OR WRITE RESPONSE FOR 88)	Yes......1<br>No......2<br>Don't know......98<br>Refused to respond......99	 ->P<br>->P<br>->P
V5	**Did any of your household members test positive for COVID-19?**<br><br>(CIRCLE ONE RESPONSE, OR WRITE RESPONSE FOR 88)	Yes......1<br>No......2<br>Don't know......98<br>Refused to respond......99	

END OF SECTION (V). GO TO SECTION (P) ON

RESPONDENT NUMBER: __ __ __ __ __ __

Food Availability (P)

INTERVIEWER TO PARTICIPANT: Getting enough food can also be a problem for some households due to coronavirus or COVID-19. I would like to ask you some questions about food availability in your household.

P	Question	Response	Skip
P1a	**Before mid-March 2020, did it ever happen that your household did not have enough food to eat?** (CIRCLE ONE RESPONSE, OR WRITE RESPONSE FOR 88)	Yes....1<br>No....2<br>Don't know....98<br>Refused to respond....99	 ->P2a<br>->P2a<br>->P2a
P1b	**How many times did your household not have enough food to eat?**	______________________<br>Don't know....98<br>Refused to respond....99	
P1c	**Why did your household not have enough to eat or not what you wanted to eat before mid-March 2020?**	Couldn't afford to buy more food....1<br>Couldn't get out to buy food....2<br>Afraid to go or didn't want to go out to buy food....3<br>Couldn't get groceries or meals delivered to me....4<br>Stores didn't have the food I wanted....5<br>Other....88<br>Don't know....98<br>Refused to respond....99	
	**Other, specify**	______________	
P2a	**Since the beginning of the COVID lockdown, has it happened that your household did not have enough food to eat?**	Yes....1<br>No....2<br>Don't know....98<br>Refused to respond....99	 ->M<br>->M<br>->M

RESPONDENT NUMBER: __ __ __ __ __ __

P2b	**During this time, how many times did your household not have enough food to eat?**	______________________<br>Don't know....98<br>Refused to respond....99	
P3	**Why did your household not have enough to eat (or not what you wanted to eat) since mid-March 2020?**<br>(SELECT ALL THAT APPLY)	Couldn't afford to buy more food....1<br>Couldn't get out to buy food....2<br>Afraid to go or didn't want to go out to buy food....3<br>Couldn't get groceries or meals delivered to me....4<br>Stores didn't have the food I wanted....5<br>Other....88<br>Don't know....98<br>Refused to respond....99	
	**Other, specify**	______________	

END OF SECTION (P). GO TO SECTION (M) ON PAGE 10

RESPONDENT NUMBER: __ __ __ __ __ __

COVID-19 Related Shocks/Coping (M)

INTERVIEWER TO PARTICIPANT: Now I would like to ask you about events that may have affected your household since mid-March.

M	Question	Response	Skip
M0	**Before mid-March of 2020, were any members of your household engaged in any of the following activities?** (PLEASE READ ALOUD AND SELECT ALL THAT APPLY)	Employment....1<br>Non-farm business operation....2<br>Farming/Agriculture operation....3<br>Don't know....98<br>Refused to respond....99	
M1	**Has your household been affected by any of these events since mid-March?** (PLEASE READ ALOUD AND SELECT ALL THAT APPLY)	Job loss....1<br>Nonfarm business closure....2<br>Disruption of farming....3<br>Disruption of livestock activities....4<br>Disruption of fishing activities....5<br>Increased price of farming or business inputs....6<br>Decreased price of farming or business outputs....7<br>Increased price of major food items consumed....8<br>Illness, injury, or death of any household member....9<br>Not affected by any listed or other major problems/events....10<br>Other....88<br>Don't know....98<br>Refused to respond....99	 <br><br><br><br><br><br><br><br><br>->Q<br>->Q<br>->Q
	**Other, specify**	______________	

CONTINUE SECTION (M) ON PAGE 11

RESPONDENT NUMBER: __ __ __ __ __ __

M2	**How did your household cope with these difficulties encountered since mid-March?**<br><br>(PLEASE DO NOT READ, SELECT ALL THAT APPLY)	Did nothing....0<br>Sale of assets....1<br>Engaged in additional income generating activities....2<br>Assistance from friends & family....3<br>Borrowed from friends & family....4<br>Took a Loan....5<br>Delayed payment obligations....6<br>Sold harvest in advance....7<br>Reduced food Consumption....8<br>Reduced nonfood Consumption....9<br>Relied on Savings....10<br>Received assistance from NGO....11<br>Took advanced payment from employer....12<br>Received assistance from government....13<br>Other....88<br>Don't know....98<br>Refused to respond....99	
	**Other, specify**	______________	

END OF SECTION (M). GO TO SECTION (Q) ON PAGE 12

RESPONDENT NUMBER: __ __ __ __ __ __

Under-five Child Healthcare Services (Q)

INTERVIEWER TO PARTICIPANT: Getting adequate health services can be a problem due to coronavirus or COVID-19. I would like to ask some questions about **children under age 5 years** your household.

Q	Question	Response	Skip
Q0	**Since mid-March 2020, were there any children under the age of 5 living in the household?**	Yes....1<br>No....2<br>Don't know....98<br>Refused to respond....99	 ->R<br>->R<br>->R
Q1	**Since mid-March, did any of the children under age 5 attend any healthcare visits?**	Yes....1<br>No....2<br>Don't know....98<br>Refused to respond....99	 ->Q3<br>->Q3<br>->Q3
Q2	**What kind of medical care did the child receive?**<br>(PLEASE DO NOT READ, SELECT ALL THAT APPLY)	Routine follow-up visits for kids....1<br>Routine vaccinations....2<br>Malaria treatment....3<br>HIV treatment....4<br>Clinic visits for any illness....5<br>Services for malnutrition....6<br>Other....88<br>Don't know....98<br>Refused to respond....99	
	**Other, specify**	______________	
Q3	**Since mid-March, was there a time you needed medical care or clinic visit for a baby or child but could not do so?**	Yes....1<br>No....2<br>Don't know....98<br>Refused to respond....99	 ->R<br>->R<br>->R
Q3a	**During this time, how many medical care or**	____________	

RESPONDENT NUMBER: __ __ __ __ __ __

	**clinical visits were missed?**		
Q4	**What kind of medical care did the child need but did not receive?** (PLEASE DO NOT READ, SELECT ALL THAT APPLY)	Routine follow-up visits for kids……1<br>Routine vaccinations……2<br>Malaria treatment……3<br>HIV treatment……4<br>Clinic visits for any illness……5<br>Services for malnutrition……6<br>Other……88<br>Don't know……98<br>Refused to respond……99	
	**Other, specify**	______________	
Q5	**Why did your child not receive healthcare?**<br><br>(PLEASE DO NOT READ, SELECT ALL THAT APPLY)	Clinic closed……1<br>Out of vaccines or medications……2<br>Did not get transportation……3<br>Lockdown……4<br>Scared to go……5<br>Other……88<br>Don't know……98<br>Refused to respond……99	
	**Other, specify**	______________	

END OF SECTION (Q). GO TO SECTION (R) ON PAGE 14

RESPONDENT NUMBER: __ __ __ __ __ __

Healthcare Services for Pregnant Women (R)

INTERVIEWER TO PARTICIPANT: Getting adequate health services can be a problem due to coronavirus or COVID-19. I would like to ask some questions about **pregnancy health services** during this time.

R	Question	Response	Skip
R0	**Is there a woman in the household who has been pregnant since mid-March 2020?**	Yes....1<br>No....2	 ->End
R1	**Since mid-March, did you attend any pregnancy-related healthcare?**	Yes....1<br>No....2<br>Don't know....98<br>Refused to respond....99	 ->R3<br>->R3<br>->R3
R2	**What kind of healthcare during pregnancy was received since mid-March?**<br><br>(PLEASE DO NOT READ, SELECT ALL THAT APPLY)	Routine antenatal visits to clinics/hospital....1<br>Clinic visits for pregnancy-related complication or concern....2<br>Delivery at clinic/hospital....3<br>C-section....4<br>Clinic visit for any illness not related to the pregnancy....5<br>Did not get medications ....6<br>Routine postnatal visit at clinic/hospital....7<br>Clinic visits for postnatal concern or complications....8<br>Other....88<br>Don't know....98<br>Refused to respond....99	
	**Other, specify**	______________	
R3	**Since mid-March, was there a time you needed medical care during the pregnancy but did not receive it?**	Yes....1<br>No....2<br>Don't know....98<br>Refused to respond....99	 ->End<br>->End<br>->End

RESPONDENT NUMBER: __ __ __ __ __ __

R3a	**During this time, how many medical care or clinical visits were missed?**	______________	
R4	**What kind of healthcare was needed but not received since mid-March**<br><br>(PLEASE DO NOT READ, SELECT ALL THAT APPLY)	Routine antenatal visits to clinics/hospital.....1<br>Clinic visits for pregnancy-related complication or concern.....2<br>Delivery at clinic/hospital.....3<br>C-section.....4<br>Clinic visit for any illness not related to the pregnancy.....5<br>Did not get medications.....6<br>Routine postnatal visit at clinic/hospital.....7<br>Clinic visits for postnatal concern or complications.....8<br>Other.....88<br>Don't know.....98<br>Refused to respond.....99	
	**Other, specify**	______________	
R5	**Why was this care not received?**<br><br>(PLEASE DO NOT READ, SELECT ALL THAT APPLY)	Clinic closed.....1<br>Out of vaccines or medication.....2<br>Did not get transportation.....3<br>Lockdown.....4<br>Scared to go.....5<br>Other.....88<br>Don't know.....98<br>Refused to respond.....99	
	**Other, specify**	______________	

SURVEY COMPLETED

RESPONDENT NUMBER: __ __ __ __ __ __

INTERVIEW END TIME: __ __: __ __ AM / PM

THE SURVEY HAS COMPLETED.

INTERVIEWER SAY:

We have completed the survey. Thank you very much for your participation, it contributes a lot to our study. We do appreciate your participation.
