# Supplementary material for: Prevalence of food insecurity amid COVID-19 lockdowns and sociodemographic indicators of household vulnerability in Harar and Kersa, Ethiopia
Source: BMC Nutr. 2024 Jan 9;10:7. doi: 10.1186/s40795-023-00815-9 (PMC10777627; doi:10.1186/s40795-023-00815-9)
Supplement: Supplementary file 1 — Additional file 1: Supplementary Methods. Wealth Index Generation. Supplementary Methods. Survey Instrument. [file 40795_2023_815_MOESM1_ESM.zip › Supplement.docx]

**Supplementary Materials**

**Food Insecurity amid COVID-19 Lockdowns: Assessing Sociodemographic Indicators of Vulnerability in Harar and Kersa, Ethiopia**

**Supplementary Methods: Wealth index generation**

A wealth index was generated based on a collection of assets and construction materials for the main dwelling of a given household. To generate the index, we followed recommendations from the DHS and the World Food Programme (WFP) that summarize steps for calculating an asset based wealth index,[49, 50] including coding instructions for Stata,[51] and an adaptation of this coding process implemented in R.[31, 48] Using these documents to guide us, we generated our wealth index by identifying a list of household assets for inclusion in our index computation, recoded all household assets into dichotomous variables; recoded dwelling materials into improved vs. non-improved dichotomous variables; divided our sample into rural and urban subsamples and assessed level of representation of a given variable within the rural and urban subsamples (per WFP recommendations, a given variable was included in further calculations if percent ownership ranged between 5 and 95 percent); employed principal components analysis with varimax rotation to calculate component scores for those households living in either rural or urban areas, which explained 45% of the variation in both subsamples; extracted and combined the PCA scores of the first component from the urban and rural subsamples; and finally organized the scores into wealth quintiles to generate a composite asset index. The resulting wealth quintiles are presented in Figure (4). The distribution of assets owned by households as well as the materials used for constructing a household’s residence are presented as percentages in Figure (5).

**Figure S1.** Household Wealth Index Distribution

**Figure S2.** Households Asset Ownership and Dwelling Construction Materials included in Wealth Index

**Supplementary Methods: Survey Instrument**
